# Optimized Hough Circle Transform for Automated Microparticle Counting in Microfluidic Platforms

**DOI:** 10.3390/mi17070819

**Published:** 2026-07-07

**Authors:** Songyuan Yan, Trevor Gerdes, Harbour Li, Timothy Morse, Lawrence Kulinsky

**Affiliations:** 1 Samueli School of Engineering, University of California, Irvine, CA 92697, USA; songyuy1@uci.edu (S.Y.); harbour@stanford.edu (H.L.); tcmorse@uci.edu (T.M.); 2 Department of Chemistry and Biochemistry, California State University, Long Beach, CA 90840, USA; trevor.gerdes01@student.csulb.edu

**Keywords:** microfluidics, Hough Circle Transform, particle detection

## Abstract

Accurate identification and enumeration of microscopic particles are important for microfluidic analysis, electrokinetic studies, and microscopy-based characterization of microfabricated systems. This study presents an optimized Hough Circle Transform (HCT) workflow for automated particle detection, sizing, and counting. Gold interdigitated electrode arrays (IDEAs) were fabricated on wafer substrates to generate electroosmotic flow, and 3 μm and 5 μm polystyrene microbeads were used as model particles. The final workflow incorporates parallelized multicore parameter optimization and composite statistical metrics based on detection accuracy and frame-to-frame standard deviation, enabling a small manually counted calibration set to be converted into locked detection parameters. In the final validation workflow, 10 manually counted calibration frames were used to optimize HCT parameters for each of four scenarios, and the locked parameters were then validated on 50 new frames per scenario (200 validation frames total) with two independent annotators. Mean validation success rates were 85.1% for 3 μm beads, 90.0% for 5 μm beads, 86.1% for 3 μm beads in mixed suspensions, and 87.2% for 5 μm beads in mixed suspensions, corresponding to object-level error rates of 17.9%, 10.9%, 19.9%, and 13.7%, respectively. Compared with the historical Generation I serial workflow, the optimized workflow reduced parameter-selection time from 24–48 h to 1–2 h, and the runtime image-processing time was approximately 45 ms per frame during offline analysis. These results show that parameter optimization is essential for robust HCT-based particle enumeration and that the workflow provides a practical analytical tool for microfluidic device characterization and electrokinetic experiments.

## 1. Introduction

Optical image analysis in microfluidic experiments is increasingly important for improving throughput, reproducibility, and precision in particle-based measurements [1,2,3,4]. Operations such as particle counting, flow visualization, and electrokinetic assembly depend on accurate interpretation of dynamic microscopy images [5,6,7,8,9,10,11,12]. Although microfluidic control has advanced substantially through improved fabrication and electrokinetic actuation [13,14,15,16,17], image analysis in many laboratory workflows still depends on manual inspection or empirically chosen thresholds. Developing robust and reproducible image processing methods is therefore important for quantitative microfluidic characterization.

In this context, particle position and apparent size provide direct physical information on flow behavior, field distribution, and assembly processes [10,11,12,18,19,20]. Among available image processing methods, the Hough Circle Transform (HCT) is particularly attractive for detecting near-circular objects in noisy images [18,19,20,21,22,23,24]. However, its performance is highly sensitive to parameter choice, and unoptimized settings can readily produce false positives, false negatives, or unstable counts across time-series image sets. To address this limitation, the present study develops a Python-based image analysis framework centered on the HCT and systematic parameter optimization for particle detection, sizing, and counting under dynamic microfluidic conditions. The underlying technique was originally introduced by Paul Hough in 1962 for recognizing complex patterns in bubble chamber photographs [21] and was subsequently generalized by Duda and Hart in 1972 to detect analytical curves, including circles and ellipses, thereby establishing the modern framework for HCT analysis [22].

Although the Hough Circle Transform is a mature image-processing method, recent microscopy and microfluidic image analysis increasingly use a broader range of particle detection, segmentation, and tracking approaches. Classical particle-tracking methods, including particle image velocimetry (PIV), particle tracking velocimetry (PTV), TrackPy, and TrackMate, provide established tools for flow measurement and trajectory reconstruction in image sequences [10,11,25,26,27]. Deep learning-based tools, including Cellpose, StarDist, and convolutional neural network detectors, have also become powerful options for microscopy image segmentation and object detection, particularly when object boundaries are irregular, overlapping, or difficult to describe using simple geometric assumptions [28,29,30].

The objective of the present study is different from these approaches. The goal is not to introduce a new fundamental circle-detection algorithm, a deep learning model, or a software package alone, but to develop a lightweight and reproducible parametric optimization workflow for HCT-based enumeration of approximately circular microparticles under controlled microfluidic imaging conditions. This positioning is important because many laboratory microfluidic experiments begin with small manually counted calibration sets rather than large, annotated training datasets. In contrast, the present workflow uses experimentally measured particle size, pixel-to-micron calibration, a limited manually counted calibration set, and a composite performance metric to identify HCT parameter combinations that provide accurate and stable particle counts across time-series images.

Compared with deep learning-based segmentation, the main advantages of the optimized HCT workflow are interpretability, low annotation demand, CPU-based deployment, and a direct connection between detection parameters and apparent particle radius. Compared with particle-tracking tools, the present workflow is more narrowly focused on rapid detection, sizing, and counting of near-circular beads rather than full trajectory reconstruction. Compared with advanced hyperparameter search strategies such as Bayesian optimization, the exhaustive grid-based search used here is computationally simple and reproducible, although Bayesian optimization may provide more efficient exploration of larger parameter spaces in future work [31]. Because these external methods were not quantitatively benchmarked in the present dataset, no claim of quantitative superiority over TrackPy, TrackMate, Cellpose, StarDist, CNN-based detectors, or Bayesian optimization is made. Accordingly, the contribution of this work should be understood as an experimentally grounded optimization and validation framework for applying HCT to microfluidic particle counting, rather than as a replacement for modern segmentation or tracking platforms. A comparison of representative particle detection and tracking approaches is summarized in Table 1.

**Figure 1:**
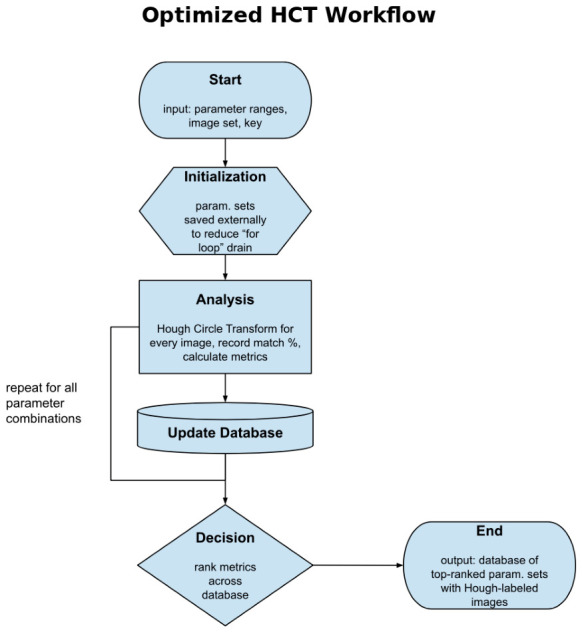
Schematic flowchart of the optimized HCT workflow. The diagram illustrates the final parameter-optimization process, which uses external parameter storage, parallel processing, and a composite accuracy-consistency metric to select locked HCT parameters for validation.

The graphical abstract illustrates the practical detection bottleneck addressed in this work: unoptimized HCT settings can generate false or missed detections, whereas optimized settings improve particle localization. Figure 1 summarizes how the final workflow uses calibration counts, composite scoring, and locked-parameter validation to address this bottleneck. The final quantitative assessment was performed using independent validation datasets, as summarized in the Results section.

The broader significance of this approach lies in analytical image processing rather than algorithmic complexity. The optimized HCT workflow provides structured, interpretable output that supports reproducible particle enumeration across changes in particle size, density, and image contrast [11,12,18,19,20,32]. Because the method is deterministic, lightweight, and directly linked to experimentally measured particle dimensions, it can be implemented as a practical preprocessing or runtime image analysis tool in electrokinetic microfluidic experiments. The framework therefore offers immediate utility for microscopy-based particle quantification and may support future closed-loop microfluidic studies in which reliable image interpretation is required.

## 2. Materials and Methods

### 2.1. Experimental

Interdigitated electrode arrays (IDEAs) were fabricated on 4-inch silicon wafers coated with a 1 μm thick thermal oxide layer (University Wafer, Inc., South Boston, MA, USA). A layer of Shipley 1827 photoresist (Shipley Company LLC, Marlborough, MA, USA) was spin-coated at 3000 rpm for 30 s using a Laurell photoresist spinner (Laurell Technologies Corporation, North Wales, PA, USA). The coated wafers were soft-baked at 95 °C for 30 min on a Dataplate PMC 732 hot plate (Barnstead Thermolyne Corporation, Dubuque, IA, USA). Photolithography was performed by exposing the wafers to UV light at an intensity of 10 mW/cm^2^ for 35 s using a Karl Suss MA6 mask aligner (SÜSS MicroTec SE, Garching, Germany) through a patterned photomask (CAD/Art Services, Inc., Bandon, OR, USA). The wafers were developed in MF-319 developer (Shipley Company LLC, Marlborough, MA, USA). A 500 Å chromium adhesion layer, deposited from 99.95% chromium granules (Kurt J. Lesker Company, Jefferson Hills, PA, USA), was applied using a CHA-600S/CV-8 thermal e-beam evaporator (Ferrotec, Livermore, CA, USA). Subsequently, a 3000 Å gold layer, deposited from 99.99% gold pellets (Kurt J. Lesker Company, Jefferson Hills, PA, USA), was added using the same system. The photoresist lift-off process was carried out in an acetone bath under ultrasonication for 5 s with a Branson CPX2800H ultrasonic cleaner (Emerson Electric Co., St. Louis, MO, USA), resulting in patterned gold IDEAs. The device architecture and related electrokinetic platform were based on our prior microfluidic study [9].

The microfluidic suspension consisted of 3 μm (0.0039 wt.%), 5 μm (0.00039 wt.%), or mixed 3 μm and 5 μm carboxyl-modified latex polystyrene beads (Invitrogen, Thermo Fisher Scientific, Waltham, MA, USA) dispersed in deionized water. A 1 mL aliquot of the original bead suspension (4 wt.%) was washed by centrifugation at 5000 rpm for 5 min using an Eppendorf centrifuge (Eppendorf SE, Hamburg, Germany). The supernatant was removed, and the pellet was resuspended in deionized water to the desired concentration. This washing and dilution procedure was repeated three times to remove residual ions from the commercial formulation.

**Figure 2:**
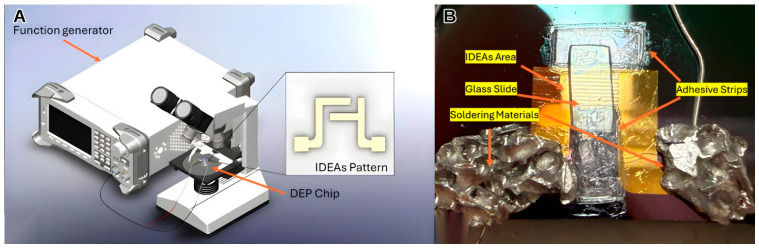
Schematic and physical illustration of the IDEA chip and experimental setup. (**A**) The zoom-in box image shows the microscale electrode pattern. The chip was connected to a function generator, and particle movement was observed and recorded through a microscope and digital camera. (**B**) Photograph of the assembled IDEA chip on a glass slide, showing the IDEA area, adhesive strips, soldering materials, and electrical connections used during the experiment.

Fabricated gold IDEA electrodes were diced into 1 cm by 1.5 cm chips, and IDEA contact pads were connected to bus wires (All Electronics Corp., Van Nuys, CA, USA) using indium solder. A double-sided adhesive tape window (3M Company, St. Paul, MN, USA) was affixed around the interdigitated region to contain the solution. Then, 4 μL of the prepared microbead suspension was pipetted into the center of the chamber and covered with a glass coverslip (Fisherbrand, Thermo Fisher Scientific, Waltham, MA, USA) to minimize evaporation and improve image focus stability. The system was observed using a Nikon Eclipse microscope with a 40× objective (Nikon Corporation, Tokyo, Japan) equipped with a SPOT RT sCMOS camera (Diagnostic Instruments, Inc., Sterling Heights, MI, USA). Images and videos were captured using SPOT 5.3/SPOT Basic acquisition software (Diagnostic Instruments, Inc., Sterling Heights, MI, USA). The experimental setup is illustrated in Figure 2.

The electrodes were connected to a function generator (Stanford Research Systems, Sunnyvale, CA, USA) to apply alternating current (AC) bias. Recordings began once the function generator was set to 5 kHz with an amplitude of 2 Vpp and 0 V DC offset. Microscope screen recordings were captured using Camtasia Recorder software 2026.1.3 (TechSmith Corporation, East Lansing, MI, USA).

The imaging experiments were conducted in bright-field mode under fixed microscope, camera, illumination, and exposure settings so that particle contrast, apparent diameter, and edge definition remained consistent across the datasets used for HCT optimization and evaluation. The recorded image frames were exported and processed offline for circle detection and particle counting. Image analysis and timing measurements were performed on a Lenovo IdeaCentre AIO 510-23ISH workstation (Lenovo, Morrisville, NC, USA) running Windows 10 (Microsoft Corporation, Redmond, WA, USA), with an Intel Core i5-6400T CPU at 2.20 GHz (Intel Corporation, Santa Clara, CA, USA), 8 GB RAM, and NVIDIA GeForce 940MX graphics with 2 GB memory (NVIDIA Corporation, Santa Clara, CA, USA). GPU acceleration was not used in the present workflow.

All image-processing scripts were executed using Python 3.14.6 (Python Software Foundation, Wilmington, DE, USA). Hough Circle Transform detection was implemented using OpenCV 4.13.0.92 through the `opencv-python` package (Open Source Vision Foundation, Palo Alto, CA, USA). Numerical calculations were performed using NumPy 2.5.0 (NumPy Developers; fiscally sponsored by NumFOCUS, Austin, TX, USA), and statistical calculations were performed using SciPy 1.18.0 (SciPy Developers; fiscally sponsored by NumFOCUS, Austin, TX, USA). The limited external-method comparison was performed using TrackPy 0.7 (TrackPy Contributors, open-source software).

Because the HCT operates on digital images, radius- and distance-related parameters are defined in pixel units rather than directly in micrometers. For the 40× imaging configuration used in this study, the 80 μm interelectrode gap corresponded to 308 pixels in ImageJ software 1.54r (National Institutes of Health, Bethesda, MD, USA), yielding an effective calibration factor of 0.260 μm/pixel. Thus, nominal 3 μm and 5 μm bead diameters correspond to approximately 11.5 and 19.2 pixels, respectively, and nominal radii correspond to approximately 5.8 and 9.6 pixels. The minimum and maximum HCT radius bounds were therefore initialized around these calibrated apparent radii and then optimized within bounded windows to account for focus, edge contrast, and apparent-radius variation.

Other HCT parameters, including dp, minDist, median blur, param1, and param2, are also image-dependent and should not be interpreted as universal physical constants. In OpenCV, dp defines the inverse ratio between the accumulator resolution and the image resolution, minDist defines the minimum allowed pixel distance between detected circle centers, median blur controls local image smoothing, param1 controls the Canny edge detector threshold, and param2 controls the accumulator threshold for circle-center detection. Radius-related parameters can be approximately rescaled after magnification changes only when pixel-size calibration is repeated, whereas threshold-related parameters generally require reoptimization after substantial changes in illumination, contrast, focus, particle density, camera resolution, exposure, or optical alignment.

To provide a representative dynamic estimate, particle displacement was visually estimated from the recorded electrokinetic videos using the 80 μm interelectrode gap as the spatial reference. The representative particle migration velocity was approximately 2 μm/s under the tested AC electrokinetic condition of 5 kHz and 2 Vpp. At this velocity, a particle would travel only approximately 0.09 μm during the 45 ms image-processing interval, indicating that particle motion was slow relative to the analysis rate and was unlikely to limit frame-by-frame particle detection under the tested conditions.

The microfluidic validation in the present study was performed at an applied electrical bias of 5 kHz and 2 Vpp. This operating point does not correspond to a single uniform bead velocity. The local bead migration speed depends on bead position within the microfluidic chamber, including distance from the interdigitated electrode edges and the local dielectrophoretic/electroosmotic flow distribution, with representative speeds of approximately 2–8 μm/s. After HCT detection parameters are optimized, the locked parameters can be used either to process previously captured images, where bead speed is not relevant because images are analyzed frame by frame, or to support live-feed analysis. Dynamic conditions are important for live-feed analysis and depend on many factors, including camera speed and the computer used for live-feed analysis. Prior live-feed use of HCT detection of electrokinetically driven microbeads [14] demonstrated that 3 μm beads were detected frame by frame using HCT, corresponding to smoothed velocities of approximately 0.5–3.2 μm/s, with short-window transient estimates below approximately 8 μm/s.

### 2.2. Methods

#### 2.2.1. HCT Detection Framework

The locations of microparticles or microbeads within a microfluidic suspension were identified from video recordings using the HCT algorithm implemented in OpenCV [33]. Once the location of each bead was determined, its movement could be tracked by comparing coordinates across consecutive frames manually or algorithmically. The HCT algorithm began by reading each input image with the OpenCV function cv.imread and converting it to grayscale. A median blur filter was then applied to smooth the grayscale image near the bead edges, reducing image noise and improving detection reliability. The grayscale image was subsequently processed to detect circular objects by analyzing gradient information at their edges. Each identified circle was represented by a set of three parameters, C (Xcenter, Ycenter, r), where Xcenter and Ycenter specify the center coordinates, and r defines the radius. Detected circles were then visualized by superimposing boundary lines on the original image.

To maintain technical consistency with OpenCV, this study adopts the library’s native parameter nomenclature. Six OpenCV HCT parameters (dp, minDist, param1, param2, minRadius, and maxRadius), together with median-blur preprocessing, governed the accuracy of the circle-detection workflow. The parameter dp is the inverse ratio of the accumulator resolution to the image resolution and was fixed at 1 in this study, ensuring identical resolutions. The parameter minDist specifies the minimum pixel distance allowed between the centers of two detected circles. A value that is too small can produce duplicate detections, while an excessively large value can cause true neighboring circles to be missed. The parameter param1 represents the Canny edge detector threshold, and param2 defines the accumulator threshold for circle-center detection. The parameters minRadius and maxRadius define the smallest and largest accepted circle radii, respectively. Because apparent bead radius can vary with focus, contrast, and manufacturing tolerance, minRadius and maxRadius were defined as bounded windows rather than single fixed values.

#### 2.2.2. Optimized Parameter-Selection Workflow

The optimized HCT workflow was designed to resolve the issues of false identification and computational inefficiency observed during preliminary serial parameter testing. The workflow schematic is presented in Figure 1. Two primary improvements were implemented. First, a secondary analysis was introduced to reduce false detections. The HCT algorithm was applied to a set of 10 manually counted calibration images, and the objective was to identify parameter sets that maintained detection rates closest to 100 percent across all images, with minimal frame-to-frame variation. Second, a refined performance metric was introduced to evaluate the consistency of detection results rather than relying solely on perfect matches. The metrics were calculated using the following relationships:

Score1 = |100 − (1/n)Σ(Pi)|(1)

Score2 = sqrt[(1/n)Σ(Pi − Pmean)^2](2)

Total Score = Score1 + Score2(3)

The accuracy parameter, designated as Score1, represents the deviation from an ideal detection rate. In Equation (1), the value 100 represents 100% detection relative to the manual count, Pi is the percentage of algorithmically identified particles relative to the manual count for frame i, and n is the total number of analyzed frames. The consistency parameter, Score2, is the standard deviation of the detection percentages across the image set. The final ranking metric, Total Score, is the sum of these two values. The optimization algorithm seeks to minimize this Total Score, thereby selecting parameter sets that provide high accuracy while maintaining stability across varying experimental frames. The theoretical minimum Total Score is 0, corresponding to an average detection percentage of 100% and a standard deviation of 0% across the analyzed frames.

To improve efficiency, the optimized program incorporated parallel processing with an eight-worker framework. The final workflow contains two distinct improvements that should be interpreted separately: the composite score improves parameter selection by penalizing both deviation from the manual count and frame-to-frame variability, whereas multicore parallelization reduces only the wall-clock time needed to evaluate the same parameter grid. A serial and a parallel evaluation of the same parameter grid using the same composite score are expected to identify the same optimum, but the parallel implementation completes the search more quickly. The parameter space was divided into eight subsets, each processed simultaneously to reduce computation time. Compared with the historical Generation I serial baseline described in Appendix A, the optimized workflow allowed task completion in 1–2 h rather than 24–48 h, depending on the size of the selected parameter space. Algorithm 1 summarizes the optimized parameter search and metric-based evaluation process, and the optimized workflow also generates output images with marked detections for visual validation. The graphical user interface used for parameter preview and software operation is documented separately in Supplementary Figure S3 and Note S1.
**Algorithm 1.** Optimized HCT parameter search with multi-objective scoringInput:  Image set I = {I1, I2, …, In}  Ground truth counts G = {G1, G2, …, Gn}  Parameter search space S:    dp in D, minDist in M, param1 in T1, param2 in T2,    minRadius in Rmin, maxRadius in Rmax Output:  Optimal parameter set P*  Detected circle sets C*  Predicted counts N*
1. Generate all candidate parameter combinations Pj in S 2. Initialize best score: Scorebest = infinity and P* = null 3. In parallel, for each candidate parameter set Pj do     a. For each image Ik in I do         i.  Read image Ik and convert it to grayscale         ii. Apply optional smoothing or denoising         iii. Apply Hough Circle Transform using Pj         iv. Record detected circle set Ck,j and count Nk,j     b. Compute Aj from predicted and reference counts     c. Compute consistency term Cj across the image set     d. Compute total score: Scorej = Aj + Cj 4. Compare all candidate scores and retain the best-performing set 5. Return P*, C*, and N*

For optimal detection accuracy, it was necessary to predefine bounded minimum and maximum particle radii before running the optimized algorithm. These radius windows were initialized from the 40× pixel-to-micron calibration and then refined through the optimization workflow. The final optimized parameter sets were generated from 10 manually counted calibration frames for each scenario and are summarized in Table 2. These values are not universal and instead reflect the optimal results obtained under the present image conditions and instrument settings.

After parameter optimization, final validation was performed as an independent locked-parameter test. For each of the four scenarios, 10 frames from the optimization experiments were used only to select the HCT parameters, and 50 additional frames from new experiments were analyzed without further parameter adjustment. Two independent annotators manually evaluated each validation frame by recording false positives and missed beads relative to the HCT output. When the two annotators differed, the final manual reference count, false-positive count, missed-bead count, correct-detection count, success rate, and error rate were calculated from the average of the two annotators’ measurements. Because these annotations were numerical frame-level count measurements rather than categorical bead identities, inter-rater agreement was assessed using a two-way absolute-agreement intraclass correlation coefficient for single measurements, ICC(2,1), rather than Cohen’s kappa. The averaged count from the two annotators was then used as the final manual reference for validation. Manual review annotations were categorized using the annotation marker legend and classification rules provided in Supplementary Figure S2 and Table S1.

To provide a comparison with an existing detection/tracking method, TrackPy [25] was applied to five representative 5 μm validation frames using the same raw images and final manual reference counts as the HCT validation. A feature diameter of 21 pixels was selected because it matched the calibrated apparent 5 μm bead diameter. The results were evaluated by frame-level count difference from the manual reference.

## 3. Results

### 3.1. Algorithmic Sensitivity and the Necessity of Optimization

A major challenge for computer vision tools such as the HCT is the avoidance of false detections, which include both false positives (nonexistent objects detected) and false negatives (real objects missed). The rate of these errors depends strongly on the choice of detection parameters. Small variations can shift outcomes dramatically, producing either no detections or thousands of false positives. This sensitivity underscores the need for automated optimization, as manual adjustment often involves a lengthy trial-and-error process.

To quantify this volatility, the algorithm was tested using a random number generator for two critical variables: the Canny edge detector threshold, param1, and the center detection threshold, param2. In a series of ten randomized trials, the unoptimized algorithm frequently failed to detect any objects, recording zero circles in seven out of ten attempts. Conversely, when detections did occur, they ranged widely from 33 to 122 circles, resulting in a low and inconsistent average of 19.2 circles per trial. These randomized trials were used only as a sensitivity analysis and not as the primary baseline comparison.

The primary non-random baseline was the original unoptimized HCT parameter set from the group’s previously used OpenCV-based analysis code associated with earlier AI-guided electrokinetic characterization work [14]. This baseline used dp = 1, minDist = 20 pixels, param1 = 48, param2 = 25, minRadius = 1 pixel, and maxRadius = 30 pixels. On a representative image containing 66 manually counted beads, this baseline detected 26 beads, corresponding to a count error rate of 60.6%.

The graphical abstract visually illustrates the performance gap between unoptimized and optimized detection. Without tuning, the algorithm produces frequent false or missed detections. In contrast, optimized parameters enable more reliable particle recognition and localization in densely populated regions. This demonstrated sensitivity supports the need for structured optimization rather than random or purely manual parameter selection.

### 3.2. Performance Improvement After Optimized Parameter Selection

To address the challenge of bridging single-frame feasibility and continuous experimental reliability, the final optimized workflow combines a composite scoring metric with multicore parallelized parameter search. Preliminary serial development work is retained in Appendix A. In the optimized workflow, candidate parameters are evaluated across calibration image sets rather than selected from a single frame, reducing false or missed detections when particle density, focus, and overlap change across frames.

The final performance values in Section 3.3 use a stricter object-level validation metric based on false positives and missed beads rather than the earlier count-only comparison. This distinction is important because absolute count error can underestimate object-level mistakes when false positives and missed detections cancel each other in the total count.

Using the historical serial baseline dynamic-image error rates as the comparison point, the final locked-parameter validation reduced the mean error rate from 46.7% to 17.9% for uniform 3 μm beads and from 35.0% to 10.9% for uniform 5 μm beads. Error-rate reduction was calculated as (baseline error rate − optimized validation error rate)/baseline error rate × 100%. Therefore, the reduction was (46.7% − 17.9%)/46.7% × 100% = 61.7% for 3 μm beads and (35.0% − 10.9%)/35.0% × 100% = 68.9% for 5 μm beads. These values replace the earlier count-only reduction estimate because the final validation counted both false positives and missed beads as object-level errors.

### 3.3. Robustness Across Particle Geometries and Mixed Populations

**Figure 3:**
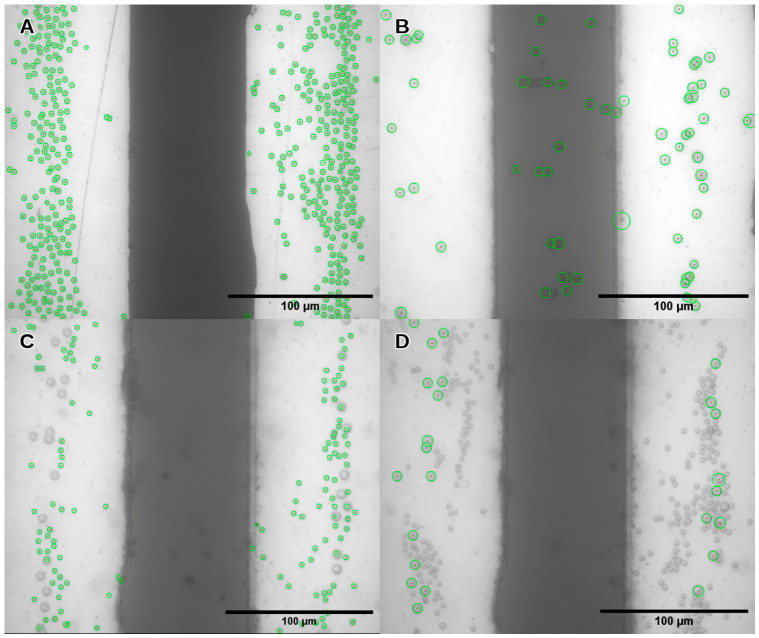
Demonstration of the optimized parameter sets generated by the optimized parameter-selection workflow. Green circular overlays indicate particles detected by the algorithm, while unlabeled particles or background features were not selected by the active parameter set. (**A**) is selected from one of the 10 calibration images for 3 μm polystyrene microbeads. (**B**) shows the detection results for 5 μm microbeads. (**C**,**D**) present the detection of 3 μm and 5 μm beads within a mixed suspension containing both particle sizes. The results indicate that the algorithm can selectively detect one particle size from a mixture.

To validate the robustness of the optimized HCT algorithm, detection performance was quantified across four experimental counting conditions: uniform 3 μm bead populations, uniform 5 μm bead populations, 3 μm beads targeted within mixed 3 μm plus 5 μm suspensions, and 5 μm beads targeted within the same mixed suspensions. Figure 3 demonstrates the ability of the optimized parameter sets to localize particles within these varying populations. Quantitative performance was evaluated primarily using the final detection error rate and the success rate relative to the manual reference counts from two independent annotators.

**Figure 4:**
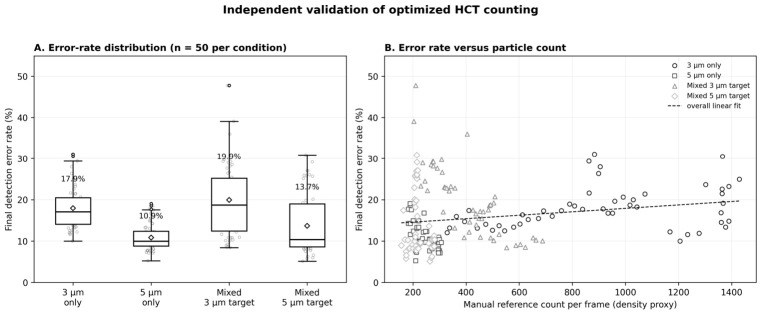
Independent 50-frame validation of optimized HCT counting under four conditions. (**A**) Box-and-whisker distributions of final detection error rates for 3 μm beads, 5 μm beads, 3 μm beads targeted in a mixed suspension, and 5 μm beads targeted in a mixed suspension (n = 50 frames per condition). Diamond markers indicate means, horizontal lines indicate medians, and overlaid points indicate individual validation frames; horizontal jitter was added only to improve visibility and does not represent an additional variable. (**B**) Detection error rate plotted against the final manual reference count per frame, used as a fixed-field-of-view density proxy. Detection error rate is shown as the primary metric because it directly captures both false-positive detections and missed beads.

The 50-frame validation results are summarized in Figure 4. The uniform 3 μm condition achieved a success rate of 85.1 ± 5.4% and an error rate of 17.9 ± 5.1%, while the uniform 5 μm condition achieved a success rate of 90.0 ± 2.8% and an error rate of 10.9 ± 3.2%. In mixed suspensions, the 3 μm target achieved a success rate of 86.1 ± 6.0% and an error rate of 19.9 ± 8.6%, while the 5 μm target achieved a success rate of 87.2 ± 6.7% and an error rate of 13.7 ± 7.4%. When the two mixed-suspension target classes were combined, the mixed-suspension validation produced an average success rate of 86.7 ± 6.3% and an object-level error rate of 16.8 ± 8.6% across 100 validation frames. Across all 200 validation frames, the overall success rate was 87.1 ± 5.7%, and the overall error rate was 15.6 ± 7.3%. Detection error rate was selected as the primary validation metric because it directly includes both false-positive detections and missed beads, whereas success rate alone can underrepresent false-positive errors.

The error rate and success rate were calculated using Equations (4) and (5):

Error rate (%) = (N_FP + N_missed)/N_manual × 100%(4)

Success rate (%) = N_correct/N_manual × 100%(5)

Manual references from the two independent annotators showed strong agreement. The ICC(2,1) values were 0.9996, 0.9697, 0.9991, and 0.9929 for the 3 μm only, 5 μm only, 3 μm mixed-suspension, and 5 μm mixed-suspension validation datasets, respectively, indicating excellent inter-rater agreement. For the mixed suspension, the reported values remain class-level size-selective counting outcomes obtained by applying separate optimized radius windows for the 3 μm and 5 μm targets; they should not be interpreted as a full bead-level size-classification confusion matrix. A true confusion matrix would require bead-level manual identity labels, including true 3 μm predicted as 3 μm, true 3 μm predicted as 5 μm, true 5 μm predicted as 3 μm, true 5 μm predicted as 5 μm, missed detections, and false positives. Object-level mixed-size classification is therefore reserved for future work.

Figure 4A summarizes the frame-level error-rate distributions for the four independent validation conditions. The uniform 5 μm bead condition showed the lowest mean error rate and the narrowest distribution, consistent with the larger pixel footprint of 5 μm beads and the greater amount of circular-edge information available for HCT detection. Under the 0.260 μm/pixel calibration, a 5 μm bead spans approximately 19.2 pixels in diameter, whereas a 3 μm bead spans approximately 11.5 pixels. In contrast, the mixed 3 μm target condition showed the highest mean error rate and broader frame-to-frame variation, indicating that smaller apparent particle size, mixed-size background objects, partial overlap, and focus-dependent edge contrast increased detection difficulty. Figure 4B further plots detection error rate against the final manual reference count per frame, which was used as a fixed-field-of-view density proxy. The overall linear fit shows only a shallow positive trend, indicating that the error rate may increase slightly with particle number, but the broad scatter also shows that density alone does not explain the remaining errors. Therefore, residual error is more likely governed by a combination of particle size, overlap, clustering, focus variation, and local image contrast rather than by particle count alone.

Residual errors are attributable to physical factors inherent to dynamic microfluidic imaging. Particle stratification in the liquid medium can cause overlapping projections. The HCT can often detect beads with minor overlap when sufficient circular edge information remains visible; however, severe overlap, bead clusters, and chain-like aggregates can obscure the boundary of individual particles and confuse edge-gradient detection. Representative failure modes include missed detections from weak or out-of-focus edges, false positives from circular debris or surface defects, duplicate detections when minDist is too small, missed neighboring particles when minDist is too large, and size ambiguity in mixed suspensions when apparent radius changes due to focus variation, projection overlap, or clustering. Representative failure-mode images for each condition are shown in Supplementary Figure S1. The supplementary figure shows examples of missed detections, false positives, duplicate detections, missed neighboring particles, and size ambiguity in mixed suspensions. To mitigate these physical interferences, electrodes were cleaned thoroughly with acetone, isopropanol, and deionized water to remove circular defects that might mimic microbeads. Suspensions were sonicated for 10 min before experiments to disrupt clusters, and microscope recordings were acquired at the highest available resolution to reduce focus-related ambiguity. The current workflow is therefore best suited to approximately circular particles with sufficient edge contrast and moderate particle density. Severe overlap, bead clusters, chain formation, and substantial focus loss remain outside the most reliable operating regime of the present HCT-based counting workflow. Future versions may combine HCT with morphological preprocessing, watershed segmentation, or lightweight learning-based segmentation to improve robustness under high-density or strongly overlapping particle conditions.

The size-calibrated TrackPy counts were approximately fourfold higher than the manual references on the five representative 5 μm validation frames. This indicates that direct use of a general localization/tracking workflow, without additional preprocessing or method-specific optimization, was not sufficient for a dense bright-field bead-counting task and supports the need for the dedicated size-calibrated HCT workflow under these conditions.

### 3.4. Computational Efficiency and Real-Time Image Analysis Potential

The gain in algorithmic precision from the optimized workflow is coupled with improved computational efficiency. The historical Generation I serial baseline described in Appendix A required 24–48 h to process the parameter space, creating a major bottleneck for experimental iteration. In contrast, the parallelized optimized workflow completes the optimization in 1–2 h. This shift concentrates the computational burden into a pre-experimental calibration phase, after which individual frames can be processed at approximately 45 ms per frame during offline image analysis. This 45 ms value represents image-processing time only and does not include camera exposure, frame transfer, disk writing, control computation, actuator response, or feedback-loop stabilization. Therefore, the present data support real-time-capable image analysis at typical microfluidic imaging rates, but they do not by themselves demonstrate complete closed-loop control.

The enhanced speed and accuracy of this HCT framework make it suitable for future extension into particle motion tracking and closed-loop-compatible microfluidic analysis. In prior work, individual particles were tracked by assigning coordinates across sequential frames and using computational control strategies to interpret their trajectories [14]. Integrating the current optimized HCT approach with electrokinetic systems may facilitate future closed-loop manipulation after additional system-level validation. A complete implementation would need to evaluate data-stream transmission delay, coordinate dropout when particles move out of the focal plane, actuation latency, and feedback-loop stability. Under the representative 5 kHz and 2 Vpp condition used here, the estimated particle displacement during 45 ms is approximately 0.09 μm, which is small relative to the apparent bead diameter; however, full closed-loop operation remains future work rather than a claim demonstrated in the present study.

## 4. Conclusions

This study demonstrates an automated and analytically useful approach for counting microscopic particles under dynamic microfluidic imaging conditions. Polystyrene microbeads were used as model particles and subjected to AC electrokinetic conditions to emulate a realistic microfluidic operating environment. By integrating the HCT with experimentally calibrated parameter optimization, the method overcomes common challenges in particle detection and quantification within microscale systems. The workflow provides tunable enumeration capability and maintains robustness across variations in image contrast, boundary definition, and particle density, enabling reliable and high-throughput measurements within the tested imaging regime.

Beyond its immediate use in microfluidic experiments, this framework provides a practical route toward reproducible image-based quantification in systems that require rapid particle recognition and counting. The deterministic workflow generates structured output that can be inspected visually, benchmarked against manual counts, and transferred across related experimental conditions after pixel-size recalibration. After final recounting, the locked optimized parameters produced an overall success rate of 87.1 ± 5.7% and an object-level error rate of 15.6 ± 7.3% across 200 independent validation frames. Future directions include object-level mixed-size classification, density-dependent performance evaluation across controlled particle concentrations, real-time particle tracking, closed-loop-compatible image analysis, and further software parallelization or distributed computing to reduce optimization time for larger parameter spaces. To facilitate broader adoption, the developed software package is made freely available at https://github.com/Songyuan-Henry-Yan/Optimized-Hough-Circle-Transform-for-Fast-and-Accurate-Microparticle-Detection-and-Counting (accessed on 5 June 2026) and archived at https://zenodo.org/records/20563945 (accessed on 5 June 2026).

## Figures and Tables

**Table 1:** Comparison of representative particle detection and tracking methods for microfluidic microscopy images.

Method	Main Advantage	Main Limitation	Best Suited Use
Manual counting	Direct visual reference	Slow and subjective	Ground truth annotation
Conventional HCT	Simple and interpretable	Sensitive to parameter choice	Circular particles under stable imaging
Optimized HCT, present study	Systematic parameter selection with low annotation demand	Parameters remain setup-dependent	Rapid counting of circular microparticles
TrackPy [25] or TrackMate [26]	Useful for particle localization and trajectory tracking	Requires preprocessing and tuning; not optimized here for size-selective counting	Particle tracking and trajectory analysis
PIV or PTV [10,27]	Strong for flow measurement and velocity-field analysis	Not mainly designed for size-selective bead counting	Microfluidic velocity analysis
Cellpose [28] or StarDist [29]	Strong segmentation capability for complex microscopy images	May require model selection, training, tuning, or more computation	Complex biological or non-circular objects
CNN-based detector [30]	High performance after suitable training	Annotation intensive and less interpretable than calibrated HCT	Large-labeled image datasets
Bayesian optimization [31]	Efficient parameter search for large spaces	Adds model and acquisition-function choices	Future acceleration of large HCT parameter searches

**Table 2:** Final optimized HCT parameter sets under the calibrated 40× imaging conditions used in this study. Radius and distance parameters are expressed in pixel units. dp is the inverse ratio between the accumulator resolution and the image resolution. Entries such as 16 or 17, 26 or 27, 180 or 181, and 66 or 67 indicate adjacent parameter values that produced indistinguishable or nearly indistinguishable scoring outcomes within the tested grid and should be interpreted as near-equivalent optima rather than uncertainty in a physical quantity.

Counting Condition	Optimized HCT Parameters
3 μm only	dp = 1; minDist = 2; median blur = 5; param1 = 16 or 17; param2 = 11; minRadius = 3; maxRadius = 7
5 μm only	dp = 1; minDist = 1; median blur = 3; param1 = 26 or 27; param2 = 28; minRadius = 7; maxRadius = 16
3 μm in 3 plus 5 μm mixture	dp = 1; minDist = 1; median blur = 3; param1 = 180 or 181; param2 = 13; minRadius = 7; maxRadius = 7
5 μm in 3 plus 5 μm mixture	dp = 1; minDist = 1; median blur = 3; param1 = 66 or 67; param2 = 13; minRadius = 12; maxRadius = 13

**Figure A1:**
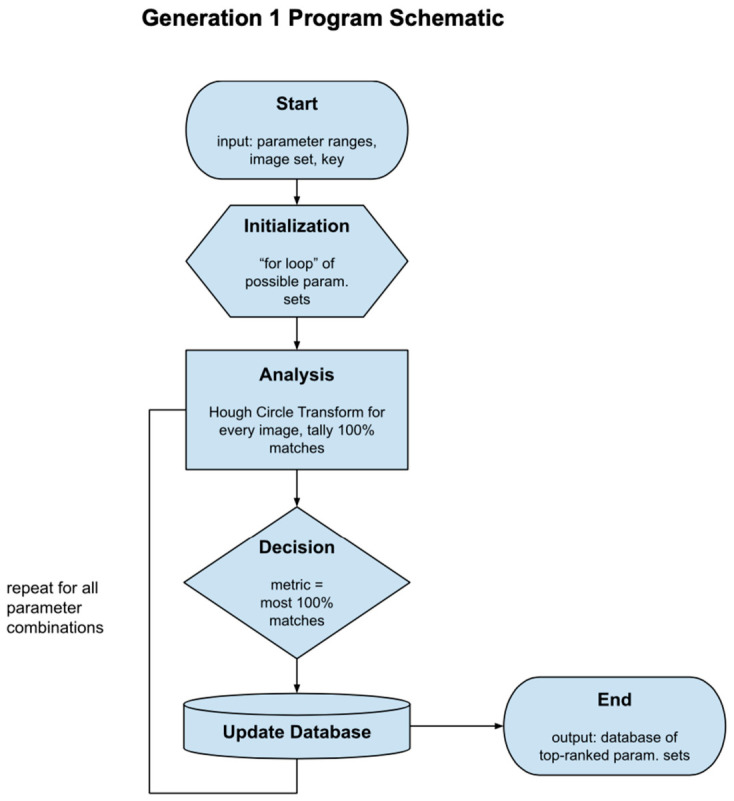
Schematic flowchart of the historical Generation I algorithm, coded in Python. The diagram illustrates the iterative “for loop” structure used to cycle through parameter combinations, utilizing a decision block to filter and store only the parameter sets that yield exact detection matches.

## Data Availability

The source code, GUI scripts, optimizer scripts, and documentation are available in the GitHub repository v3.0 (https://github.com/Songyuan-Henry-Yan/Optimized-Hough-Circle-Transform-for-Fast-and-Accurate-Microparticle-Detection-and-Counting) (accessed on 5 June 2026) and are archived at https://zenodo.org/records/20563945 (accessed on 5 June 2026).

## Supplementary Materials

The following supporting information can be downloaded at https://www.mdpi.com/article/10.3390/mi17070819/s1, Figure S1. Representative Hough Circle Transform (HCT) counting records for the four validation conditions. (a) 5 μm beads, missed = 20 and false-positive/error annotations = 3; (b) 3 μm beads in the 3 + 5 μm mixture, missed = 74 and false-positive/error annotations = 23; (c) 3 μm beads, missed = 173 and false-positive/error annotations = 24; (d) 5 μm beads in the 3 + 5 μm mixture, missed = 50 and false-positive/error annotations = 8. Red circles mark HCT detections, yellow labels mark missed beads, and green labels mark false-positive, duplicate, or size-ambiguity annotations. Figure S2. Annotation marker legend used during review of Hough Circle Transform (HCT) counting records. Red circles indicate HCT/OpenCV detections, yellow labels indicate missed beads, and green labels indicate false-positive, duplicate, or size-ambiguity annotations. Sequential numbers identify individual annotated failure events. Figure S3. Graphical user interface (GUI) used for HCT parameter preview and software operation. The interface allows the user to select a raw microscopy image; enter HCT parameters such as Canny threshold, center-detection threshold, minimum and maximum radius, minimum center-to-center distance, and median blur level; and preview detected particles before running the full optimizer. The GUI is used for visualization and parameter-range estimation; final locked parameters are selected by the optimizer using manually counted calibration frames. Note S1. Graphical user interface and software workflow. Table S1. Classification rules used for interpreting annotated HCT counting records in the supplementary material.

## Appendix A. Historical Baseline Generation I Workflow

The feasibility of this detection approach was demonstrated by developing the Generation I algorithm, whose program schematic is presented in Figure A1. The objective was to optimize parameter settings for accurate particle identification. The program first manually counted particles in 10 consecutive images taken during electroosmotic flow over a 10 min period, with one-minute intervals. It then explored all possible combinations of HCT parameters to identify those producing results most consistent with the manual counts. Consistency was defined as the ratio of beads correctly detected by the algorithm relative to the manual count. For instance, detecting 89 out of 100 beads would yield an alignment score of 0.89.

Although the Generation I algorithm successfully confirmed the feasibility of automated microparticle detection, it exhibited two main limitations. First, the simple average-based performance metric lacked the ability to identify parameter sets that were consistently effective across all images. For example, if three test images contained 100 beads each and the algorithm detected 33, 212, and 55 beads, respectively, the average would misleadingly appear correct. In this way, false positives and false negatives were not adequately filtered out. Second, the algorithm was not efficient enough to manage large hypothesis spaces containing thousands of parameter combinations. This limitation was due to the nested “for loop” structure, which in Python significantly increases computation time. Algorithm A1 summarizes the baseline workflow, in which a nested “for loop” architecture sequentially iterates through parameter ranges and applies the HCT, recording only those combinations that yielded exact detection matches. Besides the inefficiency of the code’s nested structure, this serial approach also failed to exploit multicore processing, causing a complete analysis to require one to two days. These limitations motivated the optimized workflow reported in the main text.

**Algorithm A1.** Historical baseline Hough Circle Transform workflow for microparticle counting.
Input: Image set I = {I1, I2, …, In} Radius range [rmin, rmax] HCT parameters P = {dp, minDist, param1, param2} Output: Detected circle set C for each image Particle count N for each image For each image Ik in I do 1. Read image Ik 2. Convert Ik to grayscale 3. Apply optional image smoothing or denoising 4. Apply Hough Circle Transform using: dp, minDist, param1, param2, rmin, rmax 5. Store all detected circles as Ck 6. Count the number of detected circles: Nk = size(Ck) End For Return {C1, C2, …, Cn} and {N1, N2, …, Nn}
