# Supplementary material for: Optimized Hough Circle Transform for Automated Microparticle Counting in Microfluidic Platforms
Source: Micromachines (Basel). 2026 Jul 7;17(7):819. doi: 10.3390/mi17070819 (PMC13414405; doi:10.3390/mi17070819)
Supplement: Supplementary file 1 [file micromachines-17-00819-s001.zip › micromachines-4320559-supplementary.pdf]

Supplementary Materials

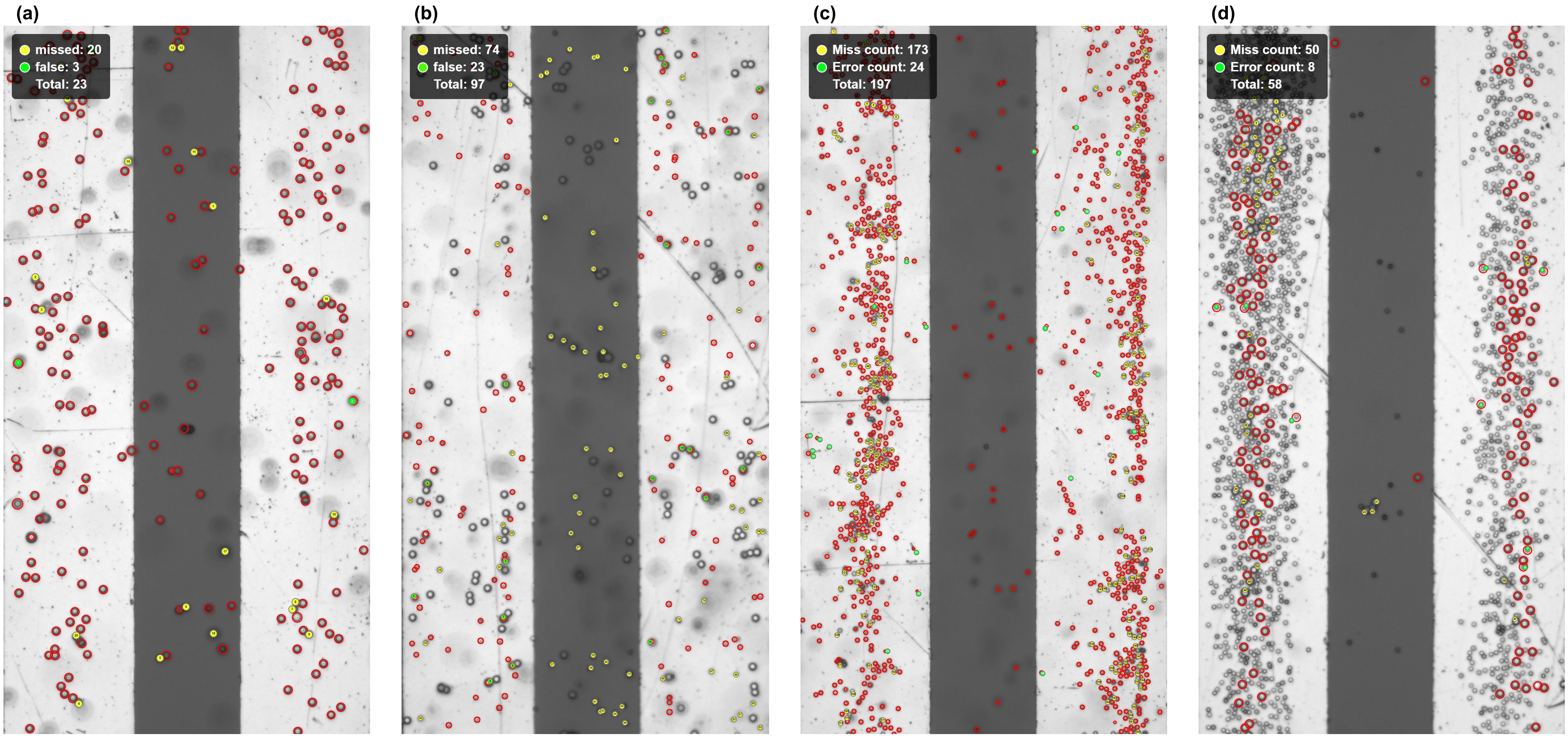

Figure S1. Representative Hough Circle Transform (HCT) counting records for the four validation conditions. (a) 5 μm beads, missed = 20 and false-positive/error annotations = 3; (b) 3 μm beads in the 3 + 5 μm mixture, missed = 74 and false-positive/error annotations = 23; (c) 3 μm beads, missed = 173 and false-positive/error annotations = 24; (d) 5 μm beads in the 3 + 5 μm mixture, missed = 50 and false-positive/error annotations = 8. Red circles mark HCT detections, yellow labels mark missed beads, and green labels mark false-positive, duplicate, or size-ambiguity annotations.

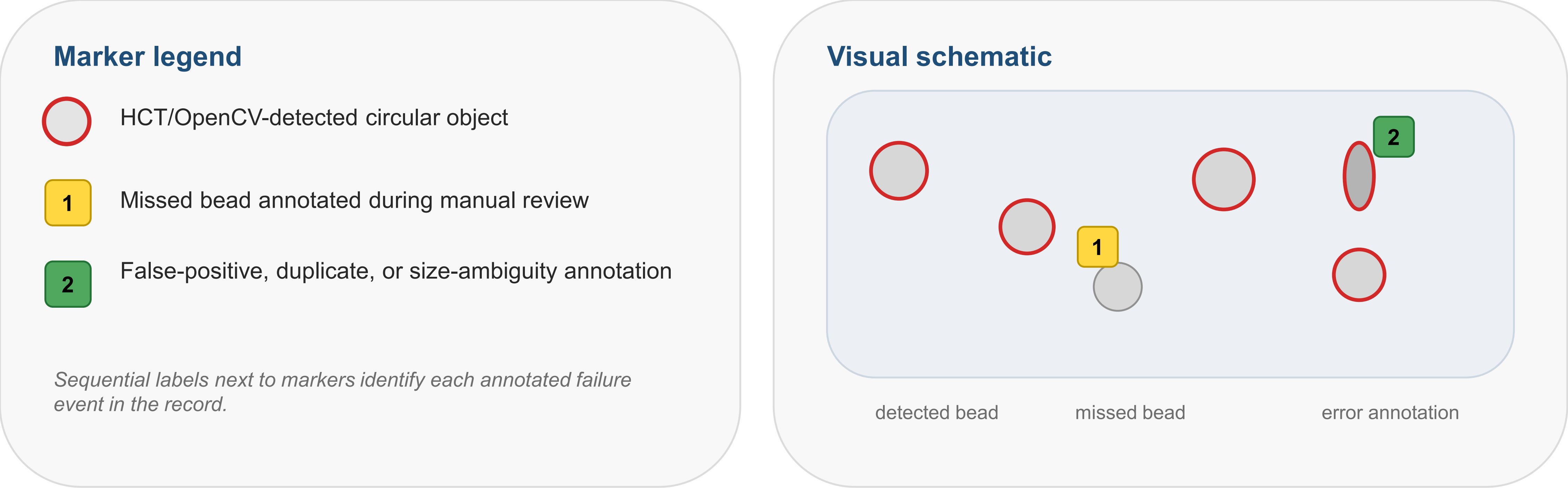

Figure S2. Annotation marker legend used during review of Hough Circle Transform (HCT) counting records. Red circles indicate HCT/OpenCV detections, yellow labels indicate missed beads, and green labels indicate false-positive, duplicate, or size-ambiguity annotations. Sequential numbers identify individual annotated failure events.

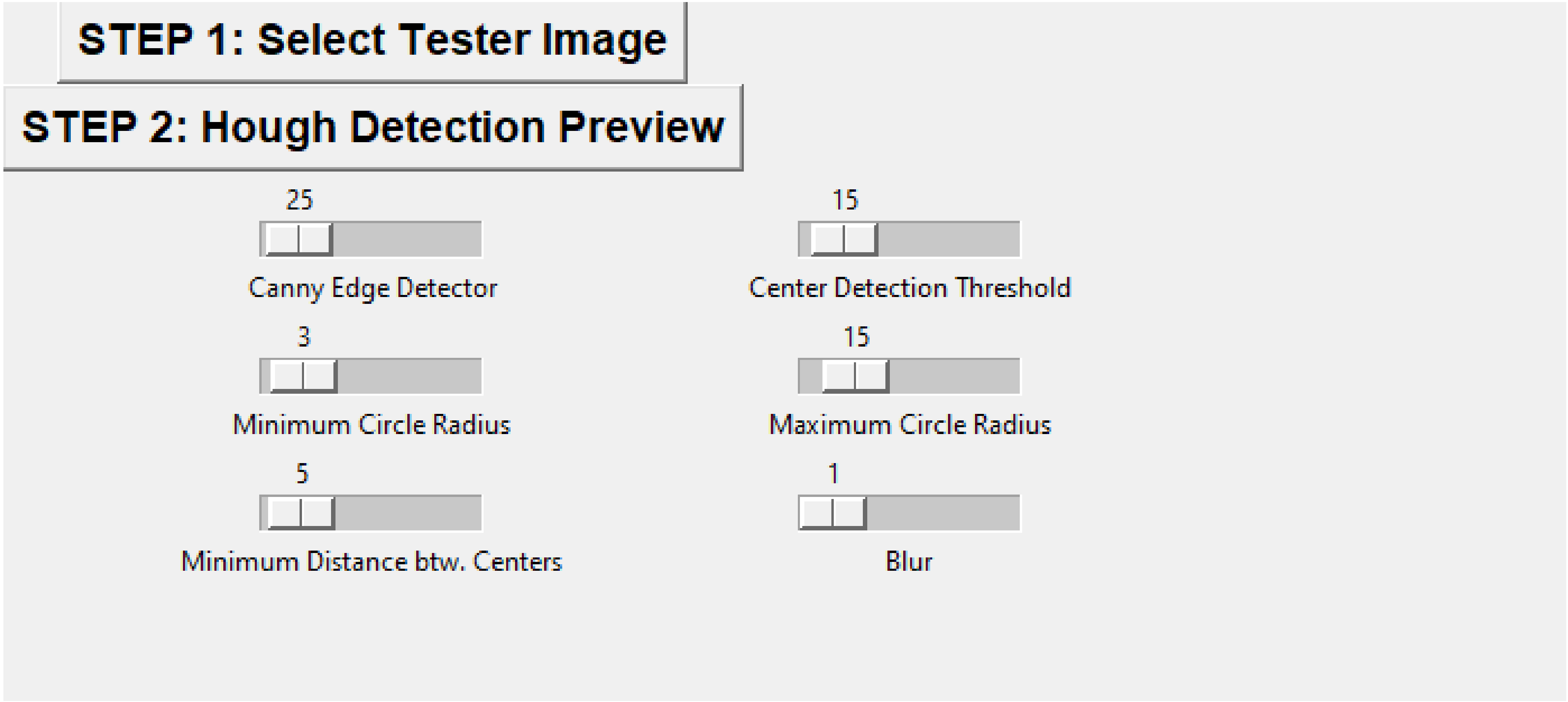

Figure S3. Graphical user interface (GUI) used for HCT parameter preview and software operation. The interface allows the user to select a raw microscopy image, enter HCT parameters such as Canny threshold, center-detection threshold, minimum and maximum radius, minimum center-to-center distance, and median blur level, and preview detected particles before running the full optimizer. The GUI is used for visualization and parameter-range estimation; final locked parameters are selected by the optimizer using manually counted calibration frames.

Supplementary Note S1. Graphical user interface and software workflow

The GUI was developed as a reproducibility and visualization tool for HCT parameter testing. It allows the user to select a raw microscopy image, adjust HCT parameters, preview detected particles, and save annotated screenshots for documentation. It does not replace the full optimization workflow; instead, it helps estimate reasonable parameter windows before optimizer-based parameter selection.

One-size workflow: for images containing one target bead size, the user selects a PNG or JPEG image, adjusts the Canny edge detector threshold, center-detection threshold, minimum radius, maximum radius, minimum center-to-center distance, and median blur level, and then runs a Hough-detection preview. Detected circles and bead counts are displayed for visual inspection.

Two-size workflow: for mixed 3 μm and 5 μm bead suspensions, two independent HCT parameter sets are applied to the same image. Separate overlay colors allow the user to visually assess whether the radius windows and threshold values preferentially detect the intended bead size.

Recommended use: approximate parameter ranges are first estimated in the GUI. These ranges are then entered into the optimizer together with calibration image filenames and manually counted bead numbers. The optimizer evaluates candidate parameter combinations across all calibration frames, ranks parameter sets using the composite scoring metric, and returns the selected locked parameter set for independent validation. Radius and distance values are pixel-based and should be recalibrated after changes in magnification, camera resolution, or cropping; threshold-related parameters may require reoptimization after changes in illumination, exposure, focus, particle density, or background contrast.

Table S1. Classification rules used for interpreting annotated HCT counting records in the supplementary material.

Failure category	Operational definition
**Missed detection**	Visible bead was present in the microscopy record, but the HCT circle overlay did not capture it.
**False-positive / duplicate error**	A detected circle corresponded to debris, a surface defect, an image artifact, duplicate localization, or another non-target circular feature.
**Missed neighboring particle**	Adjacent beads were separated manually but merged by the HCT response because edge boundaries were weak, overlapping, or too close for the chosen distance setting.
**Size-classification ambiguity**	In the 3 + 5 μm mixture, out-of-focus edges, overlapping projections, or dense local groups shifted the apparent radius enough to affect the 5 μm channel count.
**Boundary case**	Severe overlap, clusters, chain-like aggregates, or substantial focus loss required additional segmentation or manual review.
